# Origin of the selectivity differences of aromatic alcohols and amines of different *n*-alkyl chain length separated with perfluorinated C8 and bidentated C8 modified silica hydride stationary phases

**DOI:** 10.1016/j.acax.2018.100003

**Published:** 2018-12-28

**Authors:** Chadin Kulsing, Yada Nolvachai, Maria T. Matyska, Joseph J. Pesek, Joshua Topete, Reinhard I. Boysen, Milton T.W. Hearn

**Affiliations:** a Australian Centre for Research on Separation Science (ACROSS), School of Chemistry, Monash University, Melbourne, Victoria, 3800, Australia; b Department of Chemistry, San Jose State University, San Jose, CA, 95192, USA

**Keywords:** Aqueous normal-phase, Reversed-phase, Silica hydride, Shape specific separation, *n*-alkyl chain length selectivities, ACN, acetonitrile, ANP, aqueous normal-phase, BDC8, bidentate octyl, DFT, density functional theory, DH, Diamond Hydride, HILIC, hydrophilic interaction chromatography, LC, liquid chromatography, LSER, linear solvation energy relationship, PerfluoroC8, perfluorinated octyl, RP, reversed-phase

## Abstract

Perfluorinated C8-(PerfluoroC8) and bidentate anchored C8-(BDC8)-modified silica hydride stationary phases have been employed for the isocratic separation of homologous phenylalkanols and phenylalkylamines differing in their *n*-alkyl chain length, using aqueous-acetonitrile (ACN) mobile phases of different ACN contents from 10 to 90% (v/v) in 10% increments. These analytes showed reversed-phase (RP) retention behaviour with mobile phases of <40% (v/v) ACN content with both stationary phases but with the BDC8 stationary phase providing longer retention. The PerfluoroC8, but not the BDC8, stationary phase also exhibited significant retention of these analytes under conditions typical of an aqueous normal phase (ANP) mode (*i.e.* with mobile phases of >80% (v/v) ACN content), with the analytes exhibiting overall U-shape retention dependencies on the ACN content of the mobile phase. Further, these stationary phases showed differences in their selectivity behaviour with regard to the *n*-alkyl chain lengths of the different analytes. These observations could not be explained in terms of p*K*_*a*_, log *P*, molecular mass or linear solvation energy concepts. However, density functional theory (DFT) simulations provided a possible explanation for the observed selectivity trends, namely differences in the molecular geometries and structural organisation of the immobilised ligands of these two stationary phases under different solvational conditions. For mobile phase conditions favouring the RP mode, these DFT simulations revealed that interactions between adjacent BDC8 ligands occur, leading to a stationary phase with a more hydrophobic surface. Moreover, under mobile phase conditions favouring retention of the analytes in an ANP mode, these interactions of the bidentate-anchored C8 ligands resulted in hindered analyte access to potential ANP binding sites on the BDC8 stationary phase surface. With the PerfluoroC8 stationary phase, the DFT simulations revealed strong repulsion of individual perfluoroC8 ligand chains, with the perfluoroC8 ligands of this stationary phase existing in a more open brush-like state (and with a less hydrophobic surface) compared to the BDC8 ligands. These DFT simulation results anticipated the chromatographic findings that the phenylalkanols and phenylalkylamines had reduced retention in the RP mode with the PerfluoroC8 stationary phase. Moreover, the more open ligand structure of the PerfluoroC8 stationary phase enabled greater accessibility of the analytes to water solvated binding sites on the stationary phase surface under mobile phase conditions favouring an ANP retention mode, leading to retention of the analytes, particularly the smaller phenylalkylamines, *via* hydrogen bonding and electrostatic effects.

## Highlights

• Separation of phenylalkanols and phenylalkylamines of different alkyl chain length studied.
• U-shape retention dependency on organic modifier content with the perfluorinated C8 silica hydride phase observed.
• Analyte separation in both RP and ANP modes with perfluorinated C8 phase documented.
• Retention behavior of perfluorinated C8 and C8 modified silica hydride phases compared.
• Results rationalised with density functional theory simulations of the two stationary phases ligand structures.

## Graphical abstract

**Figure:**
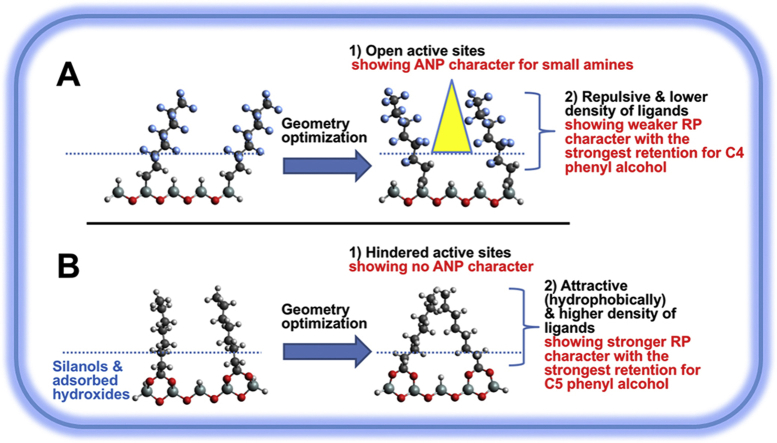

## 1 Introduction

Silanization of type-B silica with triethoxysilane results in the formation of silica hydride stationary phases with less than 5% residual silanol groups remaining on the surface [1,2]. This outcome leads to reduced irreversible adsorption of basic compounds, improved peak shapes of analytes and enhanced separation reproducibility [3,4]. Further hydrosilation of the silica hydride surface with substituted alk-1-enes or alk-1-enynes allows the introduction of various types of immobilised chromatographic ligands, broadening the range of available separation options [2] and permitting selectivity to be tuned for applications requiring reversed-phase (RP), aqueous normal phase (ANP) [4,5] or ion-exchange (IEX) [[6], [7], [8]] modes of separation. Due to their chemical stability with many aqueous-organic mobile phase compositions, these silica hydride stationary phases have been employed for separation of a wide range of compounds, such as peptides, proteins, nucleotides, nutraceuticals and medicinal compounds in pharmaceutical, food, forensic, natural product, clinical, or metabolic samples [1,2,[9], [10], [11], [12], [13], [14]].

Characterisation of the performance of silica hydride stationary phases can be achieved by a variety of different chromatographic methodologies, *e.g. via* separation of sets of compounds with different physico-chemical properties (*e.g.,* neutral, acidic and basic) under various HPLC conditions [15], and *via* deconvolution of the selectivity profiles into various analyte-stationary phase interaction clusters, such as hydrogen bonding, hydrophobic and ionic interactions as well as steric effects according to the concept of linear solvation energy relationships (LSERs) [4,7,16]. Steric selectivity effects, in particular, are complex since they are influenced by multiple analyte and stationary phase factors, including in the latter case the ligand length, extent of ligand coverage, ligand type (*i.e*., type of anchorage to the support material) and pore diameter. Such chromatographic characterisation approaches can also rely on the use of sets of homologue compounds of the same general formula, with one feature varied in a systematic manner, *e.g.* the number of functional groups or length of an alkyl chain [17,18].

This study was designed to investigate the abilities of perfluorinated octyl (perfluoroC8) and bidentate octyl (BDC8) modified silica hydride stationary phases to separate homologous phenylalkanols and phenylalkylamines under isocratic conditions with acidic (0.1% (v/v) HCOOH) mobile phases containing different ACN contents. The objective was to gain insight into the modes of separation, since these two stationary phases contain different (fluorinated *versus* non-fluorinated) functionalities, significant differences in electronegativities and dipole moments between hydrogen and fluorine atoms, different modes of immobilisation (single covalent *versus* bidentate covalent) to the silica hydride surface, yet both contain C8 moieties. The results are evaluated in terms of the dependency of analyte retention on ACN content of the mobile phase, the log *P*, p*K*_*a*_, the *n*-alkyl chain length of the analytes, linear solvation energy relationship (LSER) descriptors, and density functional theory (DFT) simulations, with these structurally discrete perfluoroC8 and BDC8 silica hydride-based adsorbents.

## 2 Materials and methods

### 2.1 Chemicals and materials

Formic acid (FA, 99% v/v) was obtained from Sigma-Aldrich (St. Louis, MO, USA). HPLC grade ACN was purchased from Merck (Darmstadt, Germany). Water was distilled and deionized with a Milli-Q system (Millipore, Bedford, MA, USA). Ammonium formate was purchased from BDH Chemicals Australia Pty. Ltd. (Kilsyth, Victoria, Australia). The PerfluoroC8 stationary phase was synthesised in house using 1H,1H, 2H-perfluoro-l-octene (3,3,4,4,5,5,6,6,7,7,8,8,8-trideca-fluoro-1-octene) and from 4.2 μm type-B silica hydride particles, 100 Å pore size, whilst the BDC8 bidentate (with two anchoring points) stationary phase, 4.2 μm particle size, 100 Å pore size, was obtained from MicroSolv Technology Corporation (Leland, NC, USA). Both stationary phases were packed into analytical columns (4.6 × 75 mm i.d.) by MicroSolv Technology Corporation (Leland, NC, USA) according to specifications that meet the requirements of industry standards.

### 2.2 Liquid chromatography

According to our previous methods [8], separations of all analytes were performed using an integrated Hewlett-Packard Series II 1100 LC system with an auto-sampler and a diode-array detector (Agilent Technologies, Palo Alto, CA, USA) operated at room temperature. ChemStation software (Agilent Technologies) was used for data acquisition. Analyte retention time (*t*_r_) was obtained at a flow rate of 0.4 mL/min under isocratic conditions with aqueous/ACN mobile phases containing different ACN contents from 10 to 90% (v/v) in 10% increments with 0.1% (v/v) formic acid. The injection volume was 5 μL and detection was performed at 210 nm and 254 nm. The retention factor (*k*) was calculated from *t*_r_ of each analyte and the column dead time *t*_0_ values (using KNO_3_ as marker) as *k* = (*t*_r_-*t*_0_)/*t*_0_. The *k* values were derived from at least duplicate measurements with the relative standard error of <1%.

### 2.3 Density functional theory based molecular simulation with Gaussian

The *ab initio* quantum chemistry program Gaussian 09 Revision E.01 (Gaussian, Inc., Wallingford, CT 06492 USA). was applied for molecular simulation of the chromatographic ligands [19]. This software was used to simulate electronic structures (*i.e.* geometry optimization) [20] which can be applied for stationary phase, analyte, and phases/analyte complexes, as well as to calculate interaction energies. Reliable results for the simulations described in this study were generated using the density functional theory (DFT) module M06-2X which is part of Minnesota hybrid meta exchange correlation functionals developed by Truhlar et al., and which includes 54% of the exchange contribution (2× represents the double amount of non-local exchange) [21,22]. The M06-2X level of DFT has been reliably applied to a wide range of H-bonding systems, such as those occurring in ionic liquids with simulation of nuclear magnetic resonance chemical shifts of analytes in different solvents [23,24]. The basis set employed in this study was cc-pVDZ, where cc-p = correlation-consistent polarized, V = valence-only basis sets, and D = double cardinal number [25]. Although an increase in cardinal number can improve the result, increasing computational resources were required [26,27].

## 3 Results and discussion

### 3.1 Effect of acetonitrile contents: separation in the RP and the ANP mode

In previous investigations by Bell et al., a type-B silica stationary phase containing immobilised fluorinated ligands was found to exhibit silanol activity similar to that observed for fused silica with strong retention of basic compounds, as well as U-shape retention dependencies of analytes on the organic modifier content in the mobile phases [28,29]. The ANP behaviour of this fluorinated type-B silica stationary phase exhibited i) major contributions from ion exchange interactions due to either ionized surface silanols or adsorbed OH^−^ ions present on the stationary phase surface (with the ion exchange mechanism resulting in retention highly dependent on mobile phase ionic strength); ii) under some conditions, the fluorinated type-B silica stationary phase exhibited characteristics analogous to cyano stationary phases with stronger retention with low ionic strength mobile phases for more positively charged compounds with higher p*K*_a_ values; and iii) contributions from weak partitioning of the analytes into water layers(s) adsorbed onto the stationary phase surface. Since a purely partition mechanism would result in retention being independent of the ionic strength of mobile phases, under some conditions, the fluorinated type-B silica stationary phase studied by Bell et al. [28,29] exhibited characteristics analogous to diol or amide stationary phases that show stronger retention for more polar compounds with smaller log D values or higher numbers of hydroxyl groups.

**Fig. 1:**
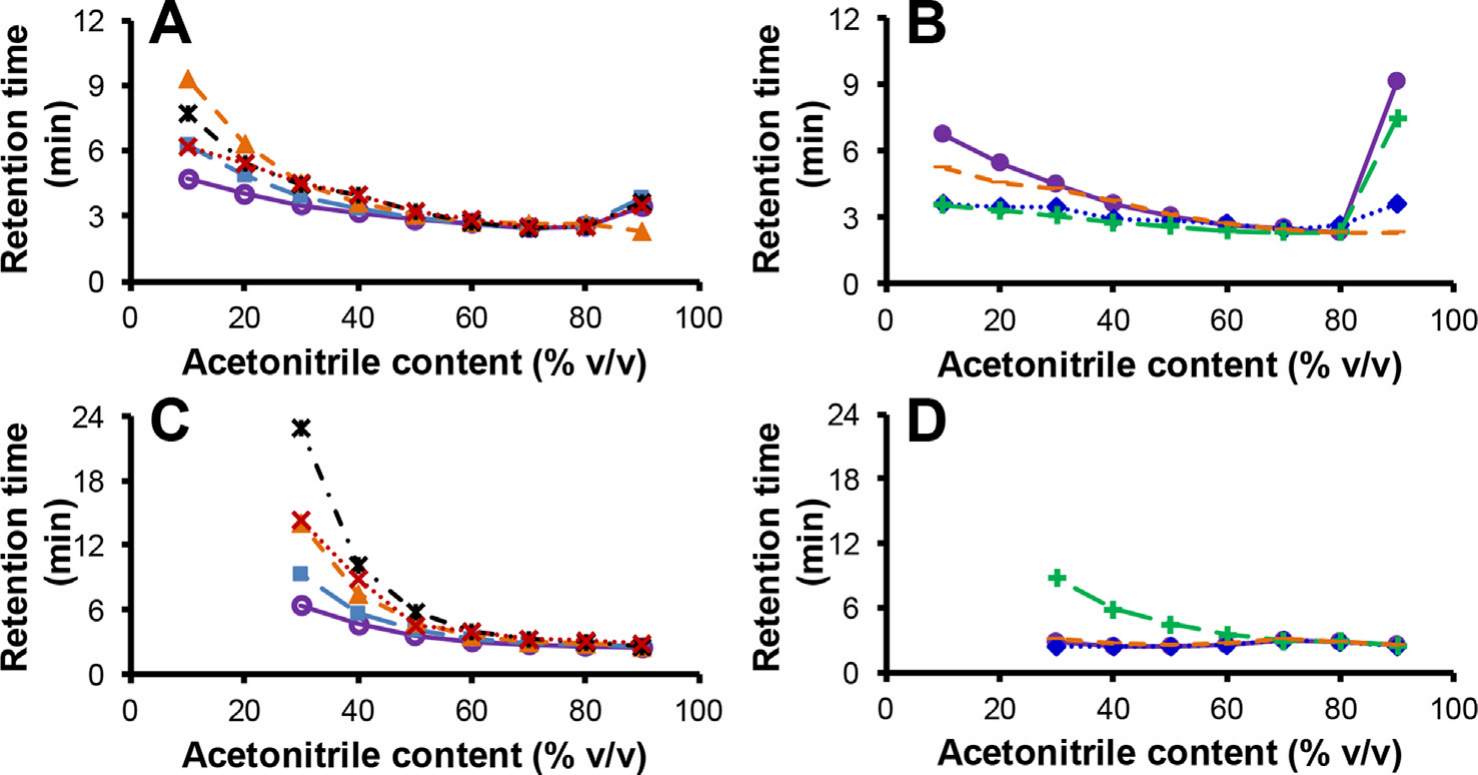
Plots of analyte retention time (min) under isocratic separation conditions as a function of mobile phase ACN content (% v/v) with 0.1% (v/v) formic acid for the PerfluoroC8 silica hydride stationary phase with the phenylalkanols (A) and phenylalkylamines (B) and for the BDC8 silica hydride stationary phase with the phenylalkanols (C) and phenylalkylamines (D). The compounds were 1-phenylethanol (**o**), 3-phenyl-1-propanol (■), 4-phenyl-1-butanol (▴), 5-phenyl-1-pentanol (), 8-phenyl-1-octanol (×), benzylamine (●), 2-phenylethylamine (♦), 3-phenylpropylamine (**+**) and 4-phenylbutylamine (−). Due to very high affinities, analyte elution did not occur with the BDC8 stationary phase with mobile phases of <30% (v/v) ACN resulting in no data plotted for these conditions in C and D.

Plots of the retention times for various phenylalkanols and phenylalkylamines (Table 1) under isocratic mobile phase conditions containing different ACN contents are shown in Fig. 1. Based on its selectivity attributes, the BDC8 silica hydride phase behaved as an archetypal RP-based stationary phase where the separation order with highly aqueous mobile phases was strongly influenced by analyte hydrophobicity. The PerfluoroC8 silica hydride also manifested this RP mode of separation with water-rich mobile phases, but exhibited an additional ANP mode of separation with mobile phases of low water content for both the phenylalkanols and the phenylalkylamines (resulting in U-shape retention dependencies with regard to the incremental increase in the organic content of the mobile phases). With the PerfluoroC8 stationary phase, the strongest retention in the RP mode was observed for 4-phenyl-1-butanol (at 10% (v/v) ACN) which also exhibited the weakest retention in the ANP mode (at 90% (v/v) ACN), Fig. 1A. In this context, the PerfluoroC8 silica hydride stationary phase behaved similar to that observed with the fluorinated type-B silica investigated by Bell et al. [28,29]. However, under acidic conditions, the PerfluoroC8 silica hydride phase, when compared to other fluorinated type-B silica stationary phases, showed a smaller contribution from ion exchange effects with some basic compounds [8,[28], [29], [30]]. Although ion exchange effects were reduced under acidic mobile phase conditions, they were still observed as shown by the retention of the positively charged amines with these silica hydride phases.

**Table 1:** Compound structures and physical properties (data obtained from http://www.chemspider.com/).

Analyte	Structure	Average mass/Da	log *P*	Polar Surface Area/Å^2^	p*K*_a_
3-phenyl-1-propanol (*n* = 3)		136.191	1.778 ± 0.183	20.2	15.04 ± 0.10
4-phenyl-1-butanol (*n* = 4)		150.218	2.321 ± 0.198	20.2	15.13 ± 0.10
5-phenyl-1-pentanol (*n* = 5)		164.244	2.851 ± 0.180	20.2	15.17 ± 0.10
8-phenyl-1-octanol (*n* = 8)		206.324	4.380 ± 0.180	20.2	15.20 ± 0.10
1-phenylethanol (*n* = 2)		122.164	1.409 ± 0.212	20.2	14.43 ± 0.20
Benzylamine (*n* = 1)		107.153	1.365 ± 0.209	26.0	9.06 ± 0.10
2-phenylethylamine (*n* = 2)		121.180	1.435 ± 0.189	26.0	9.90 ± 0.10
3-phenylpropylamine (*n* = 3)		135.206	1.910 ± 0.186	26.0	10.29 ± 0.10
4-phenylbutylamine (*n* = 4)		149.233	2.474 ± 0.185	26.0	10.66 ± 0.10

**Fig. 2:**
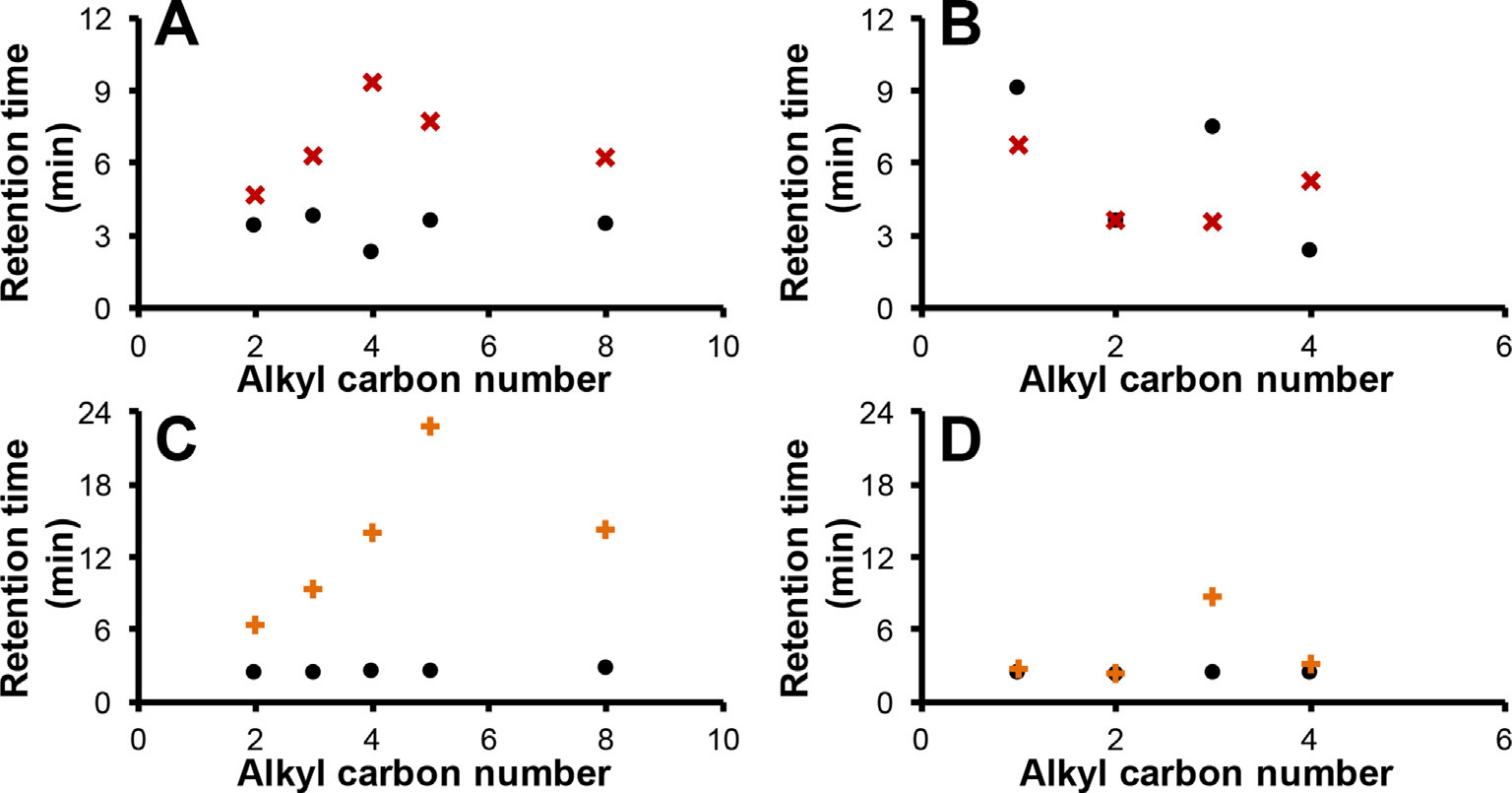
Plots of retention time (min) *vs* analyte alkyl carbon number (*n*) under isocratic separation conditions on the PerfluoroC8 silica hydride phase for phenylalkanols (**A**) and phenylalkylamines (**B**) and the corresponding plots on the silica hydride BDC8 phase for phenyl alcohols (**C**) and phenyl amines (**D**). The separation was performed under RP (×, isocratic separation at 10% (v/v) ACN on the PerfluoroC8 or +, at 30% (v/v) ACN on the BDC8 stationary phase) or ANP (•, isocratic separation at 90% (v/v) ACN on both PerfluoroC8 and BDC8 stationary phases) modes. See *n* values for all the compounds in Table 1.

These retention trends illustrate the orthogonality that exists between these two separation modes for the PerfluoroC8 silica hydride stationary phase where the most strongly retained compound in the RP mode often became the least strongly retained compound in the ANP mode. Such selectivity reversals were more clearly observed within the phenylalkanol compounds. By plotting analyte retention times *versus* their alkyl carbon numbers as shown in Fig. 2A, the inverse trend in retention in both modes of separation becomes evident (where the increasing retention in the RP mode was generally correlated with the decreasing retention in the ANP mode). Moreover, in conventional RP separations (with *e.g*., an octadecylsilica analytical column), 5-phenyl-1-pentanol and 8-phenyl-1-octanol with larger log *P* values (Table 1) show greater retention than 4-phenyl-1-butanol. In the RP mode, stronger retention is expected to correlate with larger log *P* values. However, shorter retention of 5-phenyl-1-pentanol and 8-phenyl-1-octanol was observed in comparison to 4-phenyl-1-butanol with the smaller log *P* value with the PerfluoroC8 stationary phase. This finding is indicative of a chain length dependent separation mechanism for these analytes. In comparison, the phenylalkylamines were expected to show stronger retention in comparison to phenylalkanols of similar alkyl chain length in the ANP mode for the PerfluoroC8 silica hydride stationary phase since they are more polar (cf. Table 1 for polar surface areas). Moreover, the phenylalkylamines will be strongly protonated under the investigated acidic mobile phase conditions and this has the potential to lead [8] to additional ionic interactions enhancing the retention of these amines. Thus, in the ANP mode (at 90% (v/v) ACN), the smallest amine (benzylamine) showed the strongest retention, Fig. 1B. This result again indicates that a chain length specific separation mechanism occurs with these analytes in the ANP mode. It can be further noted that benzylamine also showed stronger retention than the other amines in the RP mode.

When compared to the PerfluoroC8 stationary phase (Fig. 1A), the RP character of the BDC8 stationary phase with the phenylalkanols was more evident (see Fig. 1C and D). The largest retention in the RP mode (at 30% (v/v) ACN) was observed for 5-phenyl-1-butanol (*n* = 5, Fig. 2C). 8-Phenyl-1-octanol (*n* = 8) with the longest *n-*alkyl chain and highest log *P* value showed weaker retention than 5-phenyl-1-butanol with the BDC8 stationary phase (Fig. 1C). The 4-phenyl-1-butanol (*n* = 4) was the most retained phenylalkanol in the ANP mode with the PerfluoroC8 stationary phase (Fig. 2A). The 3-phenylpropylamine showed the greatest retention compared to the other amines in the RP mode with the BDC8 stationary phase (Fig. 1D). Interestingly, with the PerfluoroC8 stationary phase, phenylalkylamines with shorter rather than longer *n*-alkyl chains had greater retention in the RP mode.

### 3.2 Chain length specific separation effects and limitation of conventional concepts

Plots of retention time *versus* alkyl group carbon numbers (*n*) of the compounds (see Table 1 for the defined *n* values for all the compounds investigated) for the two stationary phases are shown in Fig. 2 under both the RP (**×,** for the PerfluoroC8 or **+,** for the BDC8 stationary phases) and ANP (•, for the PerfluoroC8 stationary phase) modes. These plots clearly indicate that chain length dependent separation mechanisms occur for both the phenylalkanols and the phenylalkylamines with these two silica hydride-based stationary phases. For the phenyl-alkanols, the most strongly retained compound in the RP mode with the PerfluoroC8 stationary phase occurred when *n* = 4 (Fig. 2A) whilst the corresponding observation with the BDC8 stationary phase occurred when *n* = 5 (Fig. 2C**)**. For the phenylalkylamines on the other hand, the most strongly retained compound in the ANP mode was observed when *n* = 1 and only with the PerfluoroC8 stationary phase.

**Fig. 3:**
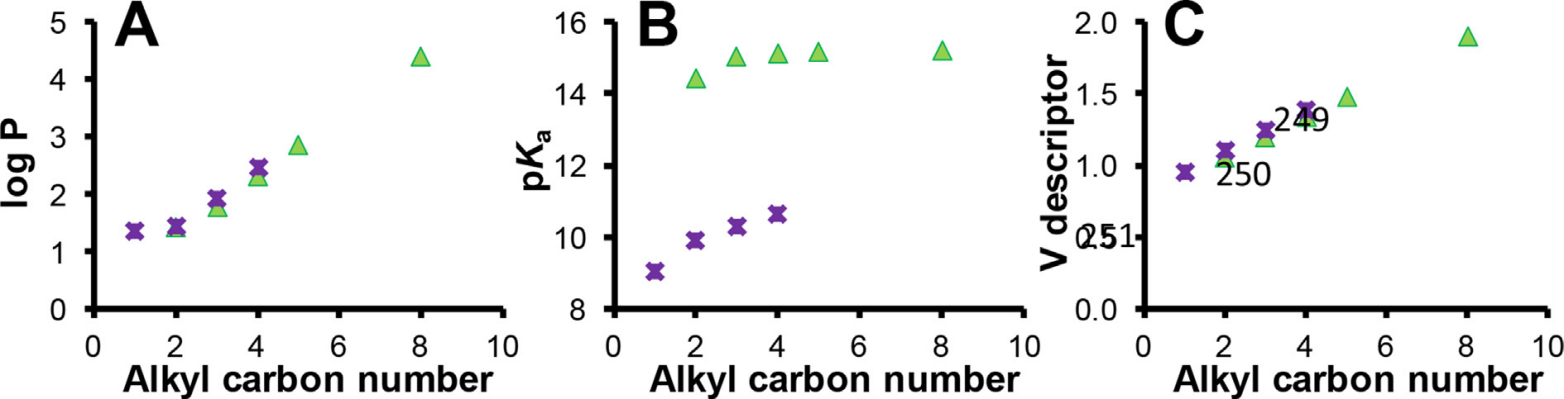
Plots of log *P* (A), p*K*_a_ (B) and V descriptor values (C) *vs* alkyl carbon number (*n*) for phenyl alcohols (▴) and phenyl amines (). See *n* values for all the compounds in Table 1.

The physicochemical properties of the studied compounds (*e.g.* log *P*, p*K*_a_ molecular mass or other LSER descriptors) varied with the *n*-alkyl chain lengths of these two sets of homologous compounds. Thus, the log *P* and p*K*_a_ values increased with *n* for the studied compounds, as shown in Fig. 3A–B (with additional physicochemical data provided in Table 1). However, the retention trends for these compounds did not follow these attributes. For example, although the intrinsic hydrophobicity increased with the length of the *n*-alkyl chains (see plots in Fig. 3A), comparison of the retention behaviour of the phenylalkanols with *n* = 2 and *n* = 8 with the PerfluoroC8 and the BDC8 stationary phases (as plotted with × and **+** in Fig. 2A and C, respectively) revealed more complex structure – retention dependencies and stationary phase effects.

Previously, the zeta-potential values of different silica hydride stationary phases, such as the PerfluoroC8, bidentate and monodentate *n*-alkyl modified silica hydride particles, have been measured in acidic media containing different acetonitrile (ACN) contents [[6], [7], [8],^30^,^31^]. Compared to the PerfluoroC8 adsorbent, more negative zeta potential values were observed for unmodified silica hydride particles as well as mono-dentate and bidentate *n*-alkyl modified silica hydride particles [31]. These findings led to the proposal that the zeta potential effect was limited spatially to a few nanometers from the stationary phase surface, a region which can be, at least in part, shielded from analytes by the presence of relatively large and/or bulky immobilised chromatographic ligands (*e.g.* phenyl groups) [7]. Thus, consideration needs to be given to the spatial organisation as well as their shielding effects of the immobilised chromatographic ligands in terms of their impact on separation selectivities with silica-hydride stationary phases operated in either the RP or the ANP modes.

Over-interpretation of the trends of log *P* and p*K*_a_ values *versus n* as shown in Fig. 3A–B could lead to an erroneously mobile phase centric conclusion that the most strongly (or weakly) retained compounds would have *n*-alkyl chain lengths of either *n* = 2 or 8, depending on whether a RP (or ANP) mode was operating. However, experimentally the largest retention under either the RP or the ANP separation mode was observed for compounds with an *n*-value that was neither 2 nor 8 for these studied stationary phases (Fig. 2A–D). As a result, the general concept that a higher pH or zeta potential value with higher ACN contents in the mobile phases results in earlier elution in either the RP or the ANP mode [7,8] cannot be applied to predict the relative retention for compounds of intermediate *n*-alkyl chain lengths as shown in Fig. 2A–D. Other established concepts such as linear solvation energy relationship (LSER) also showed no direct correlation with the retention of analytes with different *n*, as illustrated by the analyte descriptor data detailed in Table 2 and exemplified by the plot for the V descriptor values illustrated in Fig. 3C, showing a linear correlation and thus a different trend compared with any of the experimentally obtained non-linear plots shown in Fig. 2. No ANP behaviour was observed for the BDC8 stationary phase with the positively charged phenylalkylamines whilst the PerfluoroC8 stationary phase exhibited a strong ANP character with phenylalkylamines of smaller *n*-alkyl length (Fig. 1B).

**Table 2:** Compound linear solvation energy relationship (LSER) descriptors^a^ obtained from http://showme.physics.drexel.edu/onsc/models/AbrahamDescriptorsModel003.php.

Compound	E	S	A	B	V
3-Phenyl-1-propanol	0.82	0.89	0.35	0.67	1.20
4-Phenyl-1-butanol	0.81	0.90	0.33	0.70	1.34
5-Phenyl-1-Pentanol	0.80	0.90	0.33	0.72	1.48
8-Phenyl-1-octanol	0.80	0.90	0.33	0.72	1.90^a^
1-Phenylethanol	0.82	0.82	0.35	0.65	1.06
Benzylamine	0.83	0.77	0.15	0.72	0.96
2-Phenylethylamine	0.82	1.01	0.29	0.72	1.10
3-Phenylpropylamine	0.81	0.86	0.10	0.73	1.24
4-Phenylbutylamine	0.80^a^	0.97^a^	0.13^a^	0.72^a^	1.38^a^

a Approximated by assuming linear relationship between the *n*-alkyl carbon numbers and the descriptor values. **E**, Excess molar refractivity; **S**, diploarity/polarizability; **A**, hydrogen bond acidity; **B**, hydrogen bond basicity; and **V**, McGowan volume.

The above results lead to the conclusion that the chain length dependent retention behaviour of these analytes cannot be explained solely on the basis of empirical physicochemical approaches. Other concepts are required to accommodate the three dimensional molecular structures of the stationary phase, the impact of steric effects arising from the immobilisation chemistry and the role that steric hindrance of the analytes plays in their interactions with the active binding sites of the stationary phases. Conceptually, some compounds of intermediate *n*-alkyl chain length could interdigitate with immobilised ligands and thus show stronger retention, whilst others containing very long *n*-alkyl chains may be hindered from having access to the active binding sites on the stationary phase surface due to their bulky steric size.

### 3.3 Molecular modelling for explanation of the length specific effects

In our previous studies, ‘U-shape’ retention behaviour was documented for silica hydride stationary phases with considerable analyte retention observed in the aqueous normal phase mode of separation [7,8,30,32]. In principle, such retention behaviour can be expected to occur by (*i*) partitioning of polar analytes into the thin adsorbed water layer on the silica hydride phase, (*ii*) ionic interactions (especially in our case where positively charged amines can interact with PerfluoroC8 [30] and BDC8 [31] stationary phases with negative zeta potentials) or (*iii*) other non-ionic interactions of analytes with either the immobilised chromatographic ligands or the unmodified stationary phase surface, such as dipole-dipole [33], hydrophobic or hydrogen bonding interactions [[6], [7], [8],^30^,^31^], or combinations thereof. Although it has been observed for the PerfluoroC8 stationary phase [30] that polar interactions contribute to the overall retention mechanism, this type of interaction may not necessarily be the most important. If the polar or dipole-dipole type of interactions become dominant, the retention of both acidic and basic compounds (both are highly polar), would be expected to be high in the ANP mode. However, it was documented that a strong retention was only observed for basic compounds (but not acidic compounds) [30]. Since the functional group of the PerfluoroC8 silica hydride stationary phase is not acidic, it is not very likely to preferentially interact with only basic compounds. Possible explanations for the strong retention of basic compounds include the occurrence of either electrostatic interactions or hydrogen bonding interactions at a distance within a few nm of the chemically modified stationary phase surface [6,31]). Thus, the approach followed in this investigation was to give consideration to the differences of analyte access [32,33] to the surface of two types of stationary phase that are synthesised from the same silica hydride material but decorated with different chromatographic ligands.

**Fig. 4:**
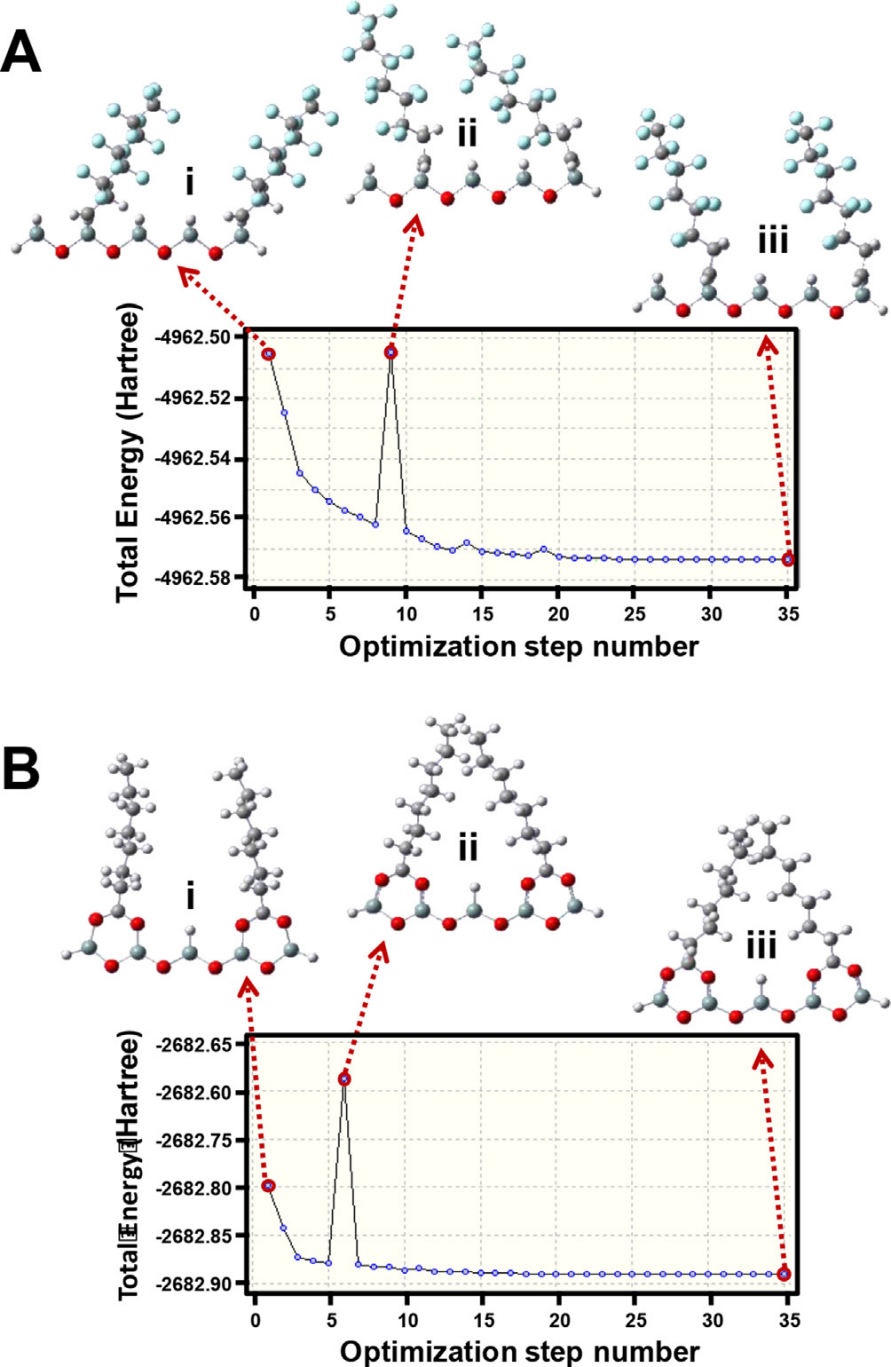
Step-by-step molecular simulation results encompassing stages (ii) to (iii) (total energy vs optimization step number) for two adjacent immobilised ligands of the PerfluoroC8 (A) and BDC8 (B) silica hydride phase on the silica hydride surface obtained by using GAUSSIAN.

**Fig. 5:**
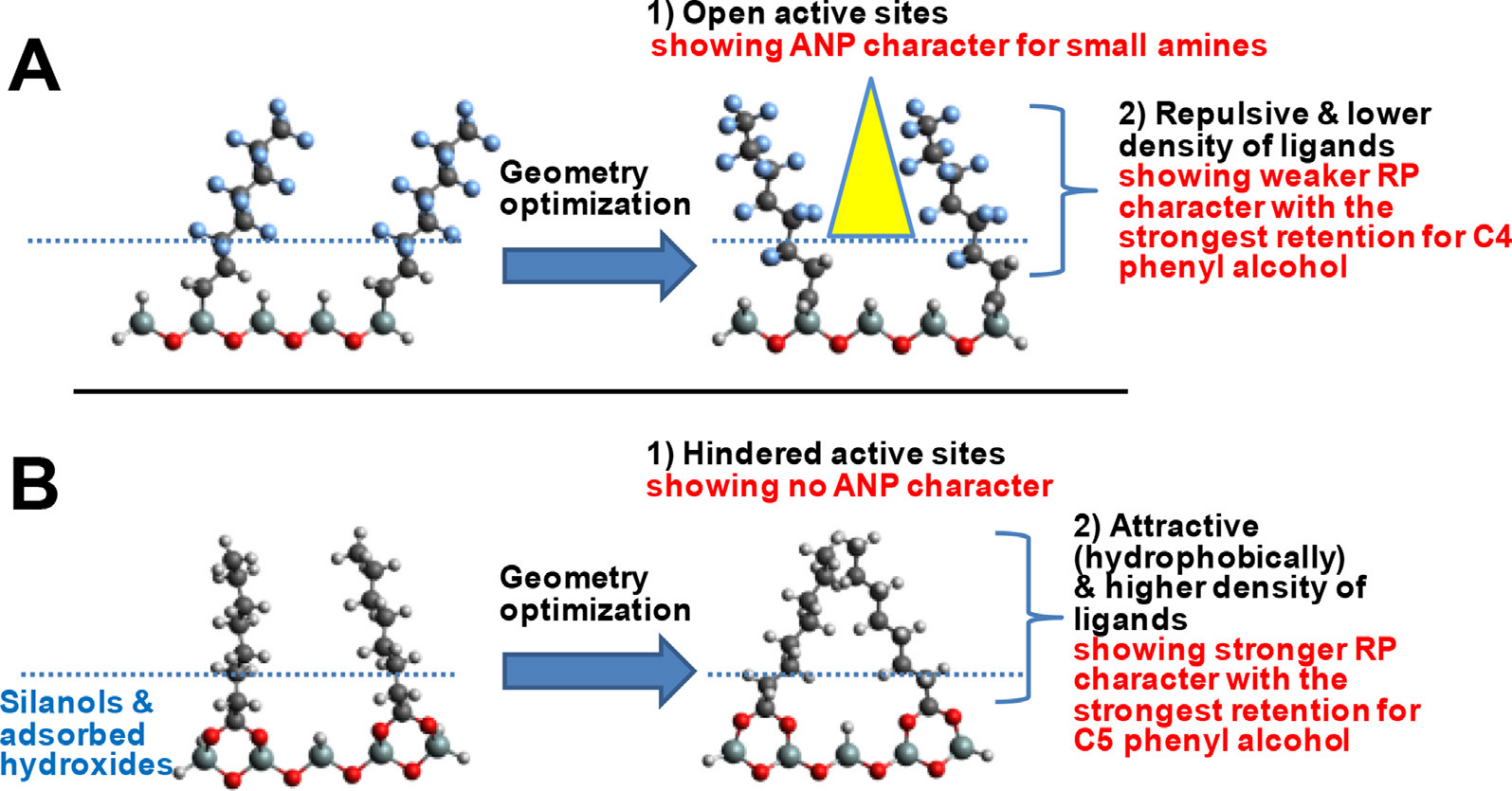
Molecular simulation results for two adjacent immobilised ligands of the PerfluoroC8 (A) and BDC8 (B) silica hydride phase on the silica hydride surface obtained with GAUSSIAN and accessibility of silica hydride surface.

One way to further examine the impact of the *n*-alkyl chain length with these compounds is to explore their conformational space using molecular dynamics simulation tools. Starting with high energy ligand geometries for adjacent unit cells of the PerfluoroC8 or BDC8 ligands immobilised onto the silica hydride surface, a step-by-step molecular simulation was carried out as depicted in Fig. 4A(i) and B(i), respectively, using the software, GAUSSIAN. The results so generated indicate that the PerfluoroC8 and BDC8 ligands have two completely different optimized low energy structures, one where the ligands repel each other and the other where the ligands are associated with one another. The implications for the accessibility of the phenylalkanols and the phenylalkylamines of different *n*-alkyl chain length due to the different geometries of the immobilised PerfluoroC8 and BDC8 ligands on the silica hydride surface are detailed in Fig. 5. Initially, a forcing routine was instigated with the immobilised PerfluoroC8 ligands resulting in the ligand moieties becoming crowded (Fig. 4A(ii)), but following repetitive step-by-step optimization it became evident that strong repulsive effects occur between individual PerfluoroC8 chains, leading to a geometry with individual PerfluoroC8 ligands being further apart (with the lowest total energy as shown in Fig. 4A(iii)). On the other hand, after the BDC8 ligands were forced initially to be close to each other (Fig. 4B(ii)), upon repetitive step-by-step optimization further attraction occurs between the individual BDC8 chains (Fig. 4B(iii)), *e.g.* hydrophobic interactions between the ligands were re-enforced, despite the bidentate nature of the anchorage.

For separations to occur in the RP mode, the open (ligand chains being further apart) or brush-like structure of the PerfluoroC8 ligands would result (locally) in binding sites of smaller hydrophobic surface area being available with this stationary phase. This outcome with ligand chain repulsions occurring with the PerfluoroC8 stationary phase (Fig. 5A), leads to weaker retention of compounds in the RP mode. Experimentally, the maximum retention was observed for the phenylalkanol with *n* = 4. On the other hand, the clustered and more hydrophobic structure of the BDC8 ligands (Fig. 5B) provides larger and more accessible hydrophobic binding regions with this stationary phase and this will result in stronger retention in the RP mode with the most strongly retained compound being the phenylalkanol with *n* = 5. The phenylalkanol compounds with *n* > 5 show weaker retention on both PerfluoroC8 and BDC8 stationary phases presumably due to their greater dynamic mobility and flexibility which results in reduced ability to interdigitate with the hydrophobic binding regions of each stationary phase.

For a silica hydride surface, the effective distance from the silica surface for binding site accessibility for retention in the ANP mode can be represented by the size of half a monolayer of water or close to the size of an OH^−^ ion [31] (as shown by the dashed lines in Fig. 5A and B). The region below each line indicates regions where analyte can (potentially) gain access to the ANP sites (with the magnitude based on previous investigations [6,31]) with each stationary phase and this can be termed the ANP active region. The more open structure of the PerfluoroC8 ligands favours the ANP mode of separation. Compared with some other silica hydride stationary phases (*e.g*., BDC8, BDC18, or undecanoic acid (UDA) modified silica hydride phase) [6,30,31], stronger retention of smaller basic compounds with the PerfluoroC8 stationary phase can be explained by greater surface accessibility to ANP active binding sites of this phase [30]. The molecular simulations shown in Fig. 5A are consistent with this conclusion.

Furthermore, since water does not preferentially solvate the fluorine atoms of the PerfluoroC8 ligand [28], a partitioning mechanism is less likely to occur. It can also be noted for the PerfluoroC8 stationary phase under the investigated acidic mobile phase conditions, any ion exchange characteristics would also be weakened [30]. As a result, the separations in the ANP mode with the PerfluoroC8 stationary phase appear to be a consequence of the participation of other separation mechanisms such as direct H-bonding processes. Other possible separation mechanisms can be considered, including solvent shielding effects where the mobile phases (eluent) cannot effectively solvate the adsorbed analytes due to the presence of PerfluoroC8 ligands. This behaviour will result in stronger retention of hydrophobic and basic compounds in both RP and ANP modes with the PerfluoroC8 stationary phase compared to an unmodified silica hydride stationary phase [7,30] but weaker retention when compared to the BDC8 stationary phase.

From the molecular simulation analysis, the analyte molecules can access to different extents ANP active binding regions of the PerfluoroC8 stationary phase [30], *e.g.* the yellow highlighted zone shown in Fig. 5A. The smallest phenylalkylamine (*n* = 1) showed the strongest retention in this mode with the PerfluoroC8 stationary phase (Fig. 2B) due to its easier access. However, with the phenylalkylamines with *n* = 2 and 3 weaker retention occurs (Fig. 2), indicating that amines with *n* > 1 cannot gain full access to the active surface (*i.e.* hindered by the increased size of the *n*-alkyl groups of these analytes leading to steric crowding with the PerfluoroC8 ligands of the stationary phase). On the other hand, the clustered organisation of BDC8 ligands shielded access to ANP active binding sites, resulting in no ANP character observed with this stationary phase (Fig. 5B). Further, the bulky phenyl group of the analytes can also sterically prevent access to these binding sites, resulting in weaker retention of the phenyalkylamines with *n* > 1 in the ANP mode (Fig. 1B).

## 4 Conclusions

These studies with homologous series of phenylalkanols and phenylalkylamines differing in their *n*-alkyl chain length with PerfluoroC8 and BDC8 stationary phases have revealed fundamental differences in separation performance and behaviour of these adsorbents. The PerfluoroC8 stationary phase exhibited retention behaviour in both the ANP and RP mode (with an U-shape dependency of the retention factors on organic modifier content with progressive increase in ACN content of the mobile phase), whilst the BDC8 stationary phase only exhibited retention in the RP mode. Analyte-dependent chain-length dependent separations were also demonstrated with these silica hydride-based stationary phases. However, unlike conventional RP separations, where retention increases with compounds as the substituent *n-*alkyl chain length increases, with the PerfluoroC8 stationary phase analyte-dependent chain length dependent effects resulted in the strongest retention with phenylbutanol but for the BDC8 stationary phase this occurred with phenylpentanol. Retention consistent with an ANP mode of separation was only observed with the smaller phenylalkylamines with the PerfluoroC8 stationary phase.

Density functional theory (DFT) simulations revealed different optimized ligand structures for the two stationary phases. The BDC8 ligands was found to exist in a much more clustered organisation leading to a shielded surface with no ANP behaviour observed. This clustered structure promoted analyte binding with the active hydrophobic surface, allowing stronger retention of the phenylalkanols with the BDC8 stationary phase in the RP mode. On the other hand, the isolated, brush-like structure of the perfluoroC8 ligands leads to uncovered/exposed regions of the stationary phase allowing access to the stationary phase surface and enabling the ANP mode of separation to occur with the small phenylalkylamines. However, these interactions with the PerfluoroC8 ligands appear to be weaker that for BDC8 stationary phase leading to phenylbutanol being more strongly retained than phenylpentanol as was found with the BDC8 stationary phase.

## Declaration of interests

None.

## References

[1] Pesek J.J., Boysen R.I., Hearn M.T.W., Matyska M.T. (2014). Hydride-based HPLC stationary phases: a rapidly evolving technology for the development of new bio-analytical methods. Analytical Methods.

[2] Pesek J.J., Matyska M.T., Boysen R.I., Yang Y., Hearn M.T.W. (2013). Aqueous normal-phase chromatography using silica-hydride-based stationary phases. TrAC, Trends Anal. Chem..

[3] Pesek J.J., Matyska M.T. (2010). Silica hydride--chemistry and applications. Adv. Chromatogr..

[4] Soukup J., Janas P., Jandera P. (2013). Gradient elution in aqueous normal-phase liquid chromatography on hydrosilated silica-based stationary phases. J. Chromatogr. A.

[5] Boysen R.I., Yang Y., Chowdhury J., Matyska M.T., Pesek J.J., Hearn M.T.W. (2011). Simultaneous separation of hydrophobic and hydrophilic peptides with a silica hydride stationary phase using aqueous normal phase conditions. J. Chromatogr. A.

[6] Kulsing C., Yang Y., Boysen R.I., Matyska M.T., Pesek J.J., Hearn M.T.W. (2015). Role of electrostatic contributions in the separation of peptides with silica hydride stationary phases. Analytical Methods.

[7] Kulsing C., Yang Y., Munera C., Tse C., Matyska M.T., Pesek J.J., Boysen R.I., Hearn M.T.W. (2014). Correlations between the zeta potentials of silica hydride-based stationary phases, analyte retention behaviour and their ionic interaction descriptors. Anal. Chim. Acta.

[8] Kulsing C., Yang Y., Matyska M.T., Pesek J.J., Boysen R.I., Hearn M.T.W. (2015). Prediction of the zeta potentials and ionic descriptors of a silica hydride stationary phase with mobile phases of different pH and ionic strength. Anal. Chim. Acta.

[9] Pesek J.J., Matyska M.T. (2006). Silica hydride surfaces: versatile separation media for chromatographic and electrophoretic analyses. J. Liq. Chromatogr. Relat. Technol..

[10] Nolvachai Y., Kulsing C., Boysen R.I., Matyska M.T., Pesek J.J., Marriott P.J., Hearn M.T.W. (2014). Comparison of the performance of different silica hydride particles for the solid-phase extraction of non-volatile analytes from dark chocolate with analysis by gas chromatography-quadrupole mass spectrometry. Food Chem..

[11] Pesek J.J., Matyska M.T. (2005). Hydride-based silica stationary phases for HPLC: fundamental properties and applications. J. Separ. Sci..

[12] Pesek J.J., Matyska M.T., Hearn M.T.W., Boysen R.I. (2009). Aqueous normal-phase retention of nucleotides on silica hydride columns. J. Chromatogr. A.

[13] Pesek J.J., Matyska M.T. (2009). Our favorite materials: silica hydride stationary phases. J. Separ. Sci..

[14] Pesek J.J., Matyska M.T. (2012). A new approach to bioanalysis: aqueous normal-phase chromatography with silica hydride stationary phases. Bioanalysis.

[15] Yang Y., Boysen R.I., Kulsing C., Matyska M.T., Pesek J.J., Hearn M.T.W. (2013). Analysis of polar peptides using a silica hydride column and high aqueous content mobile phases. J. Separ. Sci..

[16] Wilson N.S., Nelson M.D., Dolan J.W., Snyder L.R., Wolcott R.G., Carr P.W. (2002). Column selectivity in reversed-phase liquid chromatography: I. A general quantitative relationship. J. Chromatogr. A.

[17] Begnaud F., Larcinese J.P., Fankhauser P., Maddalena U. (2016). LogP measurement of a highly hydrophobic properfume: evaluation of extrapolation of RP-HPLC results and impact of chemical moieties on accuracy. Flavour Fragrance J..

[18] Steimer S.S., Kourtchev I., Kalberer M. (2017). Mass spectrometry characterization of peroxycarboxylic acids as proxies for reactive oxygen species and highly oxygenated molecules in atmospheric aerosols. Anal. Chem..

[19] Frisch M.J., Trucks G.W., Schlegel H.B., Scuseria G.E., Robb M.A., Cheeseman J.R., Scalmani G., Barone V., Mennucci B., Petersson G.A., Nakatsuji H., Caricato M., Li X., Hratchian H.P., Izmaylov A.F., Bloino J., Zheng G., Sonnenberg J.L., Hada M., Ehara M., Toyota K., Fukuda R., Hasegawa J., Ishida M., Nakajima T., Honda Y., Kitao O., Nakai H., Vreven T., Montgomery J.A., Peralta J.E., Ogliaro F., Bearpark M.J., Heyd J., Brothers E.N., Kudin K.N., Staroverov V.N., Kobayashi R., Normand J., Raghavachari K., Rendell A.P., Burant J.C., Iyengar S.S., Tomasi J., Cossi M., Rega N., Millam N.J., Klene M., Knox J.E., Cross J.B., Bakken V., Adamo C., Jaramillo J., Gomperts R., Stratmann R.E., Yazyev O., Austin A.J., Cammi R., Pomelli C., Ochterski J.W., Martin R.L., Morokuma K., Zakrzewski V.G., Voth G.A., Salvador P., Dannenberg J.J., Dapprich S., Daniels A.D., Farkas Ö., Foresman J.B., Ortiz J.V., Cioslowski J., Fox D.J. (2009). Gaussian 09.

[20] Liu B., McLean A.D. (1973). Accurate calculation of the attractive interaction of two ground state helium atoms. J. Chem. Phys..

[21] Zhao Y., Truhlar D.G. (2008). The M06 suite of density functionals for main group thermochemistry, thermochemical kinetics, noncovalent interactions, excited states, and transition elements: two new functionals and systematic testing of four M06-class functionals and 12 other functionals. Theor. Chem. Acc..

[22] Zhao Y., Truhlar D.G. (2011). Applications and validations of the Minnesota density functionals. Chem. Phys. Lett..

[23] Chesman A.S.R., Hodgson J.L., Izgorodina E.I., Urbatsch A., Turner D.R., Deacon G.B., Batten S.R. (2014). Anion–anion interactions in the crystal packing of functionalized methanide anions: an experimental and computational study. Cryst. Growth Des..

[24] Chen S., Vijayaraghavan R., MacFarlane D.R., Izgorodina E.I. (2013). *Ab initio* prediction of proton NMR chemical shifts in imidazolium ionic liquids. J. Phys. Chem. B.

[25] Dunning T.H. (1989). Gaussian basis sets for use in correlated molecular calculations. I. The atoms boron through neon and hydrogen. J. Chem. Phys..

[26] Mezei P.D., Csonka G.I., Ruzsinszky A. (2015). Accurate complete basis set extrapolation of direct random phase correlation energies. J. Chem. Theor. Comput..

[27] Okoshi M., Atsumi T., Nakai H. (2015). Revisiting the extrapolation of correlation energies to complete basis set limit. J. Comput. Chem..

[28] Bell D.S., Cramer H.M., Jones A.D. (2005). Rational method development strategies on a fluorinated liquid chromatography stationary phase: mobile phase ion concentration and temperature effects on the separation of ephedrine alkaloids. J. Chromatogr. A.

[29] Bell D.S., Jones A.D. (2005). Solute attributes and molecular interactions contributing to "U-shape" retention on a fluorinated high-performance liquid chromatography stationary phase. J. Chromatogr. A.

[30] Kulsing C., Yang Y., Sepehrifar R., Lim M., Toppete J., Matyska M.T., Pesek J.J., Boysen R.I., Hearn M.T.W. (2016). Investigations into the separation behaviour of perfluorinated C8 and undecanoic acid modified silica hydride stationary phases. Anal. Chim. Acta.

[31] Kulsing C., Nolvachai Y., Marriott P.J., Boysen R.I., Matyska M.T., Pesek J.J., Hearn M.T.W. (2015). Insights into the origin of the separation selectivity with silica hydride adsorbents. J. Phys. Chem. B.

[32] Yang Y., Matyska M.T., Boysen R.I., Pesek J.J., Hearn M.T.W. (2013). Simultaneous separation of hydrophobic and polar bases using a silica hydride stationary phase. J. Separ. Sci..

[33] Armstrong D.W., Tang Y., Chen S., Zhou Y., Bagwill C., Chen J.-R. (1994). Macrocyclic antibiotics as a new class of chiral selectors for liquid chromatography. Anal. Chem..

